# Practical guidance for navigating buprenorphine discontinuation

**DOI:** 10.1186/s12954-025-01353-2

**Published:** 2025-12-05

**Authors:** Kento Sonoda, Amanda B. Hilmer, Jennifer K. Bello

**Affiliations:** Department of Family and Community Medicine, Saint Louis University School of Medicine, St. Louis, MO USA

**Keywords:** Buprenorphine, Opioid use disorder, Overdose

## Abstract

Approximately 4.8 million people in the United States had opioid use disorder in 2024, with more than one hundred thousand experiencing an overdose death. Despite the established benefits of buprenorphine on morbidity and mortality, many patients may express an interest in discontinuing their medications for various reasons, including stigma surrounding addiction. There are limited studies describing how to navigate buprenorphine discontinuation. This commentary provides a practical approach to navigating buprenorphine discontinuation, using two case studies. Ideally, clinicians will develop a deep understanding of the patient’s substance use journey, allowing them to understand both the internal and external factors driving their decision. Through this understanding, a clinician can develop a plan based on their well-being, safety, and risk factors for return to use. It is vital to reinforce that buprenorphine is a life-saving medication, can be continued life-long, and is a tool for recovery, not a barrier.

## Introduction

In the United States, 4.8 million people aged 12 or older had opioid use disorder (OUD), and more than one hundred thousand drug overdose deaths occurred in 2024 [1, 2]. Buprenorphine is a partial mu-opioid receptor agonist and a kappa antagonist, and buprenorphine-naloxone has been widely used to treat OUD by alleviating physical withdrawal symptoms and suppressing cravings since its approval in 2002.

Despite the established benefits of buprenorphine on morbidity and mortality along with financial costs, many patients may express their interest in discontinuing their medications for various reasons [3–6]. First, stigma around addiction negatively affects individuals with OUD from both linkage to care and retention in care [7]. Thus, patients on buprenorphine may implicitly desire to discontinue their treatment to distance themselves from stigmatizing circumstances. Secondly, patients on buprenorphine may face a conflicting treatment philosophy of 12-step meetings which do not traditionally support medications for OUD, and experience pressure to discontinue buprenorphine to become “completely sober” from their addiction [8]. Additionally, some patients may wish to stop buprenorphine because it can lead to physical dependence and then necessitate regular office visits for refills, may complicate extended travel plans, and can be impacted by lapses in insurance coverage. Lastly, patients may desire to discontinue buprenorphine because of potential negative impacts on their employment although the Americans with Disabilities Act protects people in recovery from OUD [9, 10].

There is no definite evidence on the treatment duration of buprenorphine. In fact, a multicenter cohort study showed that treatment duration was not associated with all-cause mortality and drug overdose in regression analyses [11]. Given the remarkable clinical benefit to patients with OUD, the American Society of Addiction Medicine clinical consideration article recommends clinicians keep prescribing buprenorphine as long as patients benefit, which can be lifelong [12]. Each patient with OUD has their own unique story related to their addiction, making it essential that clinicians individualize treatment plans by respecting patient desires while also ensuring patient safety. There are limited studies and established guidelines describing how to navigate buprenorphine discontinuation, although there is growing evidence on how to initiate buprenorphine in various ways [12, 13]. Our article aims to provide a practical approach to navigating buprenorphine discontinuation using case studies.

## Case 1

Case vignette: 42 year-old cis-gender female patient with severe OUD presents to your clinic as a regular follow up visit. She was first prescribed buprenorphine-naloxone 8–2 mg films twice a day three months ago. She used to take 4 capsules of fentanyl 2 mg every day prior to the treatment initiation. Although buprenorphine-naloxone helps her craving, the patient intermittently takes a capsule of fentanyl 2 mg along with methamphetamine, up to four times per week, over the past three months. She reports her last use of fentanyl (a capsule) and methamphetamine (“just a little”) was yesterday. The urine drug screen in the office is positive for fentanyl, methamphetamine, and buprenorphine. The patient states she is interested in tapering off buprenorphine.

This patient is unstable in her drug use and has a desire to stop taking buprenorphine-naloxone placing her at high risk of return to consistent use of fentanyl and methamphetamine. The clinician should consider asking the following questions to better understand the patient’s circumstances and rationale for stopping buprenorphine-naloxone in addition to identifying both internal and external resources an individual can utilize in their recovery, known as recovery capital [14]. An individual’s recovery capital consists of resources at the individual, interpersonal, and community level.

– “What are your thoughts about buprenorphine-naloxone? Do you have any concerns?”
– “What is your understanding of buprenorphine-naloxone treatment?”
– “What made you interested in discontinuing buprenorphine-naloxone?”
– “Tell me about the circumstances of your current fentanyl and methamphetamine use patterns?”
– “Do you have cravings and/or withdrawal symptoms on your current dose of buprenorphine-naloxone?”
– “What are your goals for substance use if you were to stop using buprenorphine-naloxone?”
– “Are there any potential triggers of relapse or return to use?”
– “Are you on individual and/or group therapy?”
– “Have you tried to taper off buprenorphine-naloxone by yourself in the past? If so, how did it go?”
– Recovery capital questions^14^:
o “Who do you turn to when you need help with your recovery?”
o “What strategies do you use to manage cravings or urges?”
o “How do you handle stress without turning to substances?”
o “What are your reasons for wanting to stay sober?”
o “How committed are you to your recovery journey?”
o “How would you rate your overall physical health?”
o “Do you have any mental health concerns that need to be addressed?”
o “Do you have stable housing? Can you tell me about the people you stay with? What is your relationship with this person/people? Can you tell me about their drug and alcohol use?”
o “Are you currently employed?”
o “Do you have access to resources for basic needs?”

After further discussion, the patient explains she is staying “here and there” with friends who us substances and cannot move back in with a family member until she has stopped using illicit drugs completely, leaving her without a support network [15]. The patient also expresses significant challenges related to triggers due to her housing instability despite her desire to achieve and maintain sobriety. The patient explains she continues to have cravings on the current dose of buprenorphine-naloxone 16–4 mg. Without her family or other support network, she deals with cravings by using both small amounts of fentanyl and methamphetamine. She is unemployed, having lost her job six months ago due to drug use. She denies any physical health problems but does report struggling with significant depression and anxiety as she works through her conflicting desire to stay sober and her return to use to deal with the reality of her current situation, being isolated and without the ability to meet her basic needs. In this case, clinicians consider an increased dose of buprenorphine-naloxone to 24–6 mg daily to help with cravings. Clinicians should acknowledge and congratulate the patient on her motivation to enter recovery and her resilience in navigating a difficulty housing situation. Housing assistance should be discussed, including recovery housing/sober living to help the patient remove herself from the risky situation where she may live with friends who are actively using substances. The clinician should also connect the patient to a behavioral health specialist to assess her mental health needs as well as a peer support specialist to serve as a contact for support as she works toward recovery. Finally, clinicians discuss ways to make receiving care and picking up prescriptions more convenient. For example, offering telehealth visits with urine drug screens to be done at a lab location that is convenient for the patient. Clinicians discuss the option of medications being delivered rather than having to be picked up.

If, after a thorough discussion, including an explanation of the risks associated with tapering off buprenorphine-naloxone while the patient is facing numerous barriers to achieving stability in her recovery, the patient continues to desire taper from the medication, the clinicians should collaborate with the patient to create a personalized tapering plan. Importantly, even after tapering off buprenorphine-naloxone, continued aftercare remains a critical component of the recovery process. The tapering plan should include implementation of harm reduction strategies to minimize the chance of continued use and overdose. Examples of such strategies include:

1. Encouraging the patient to keep several buprenorphine-naloxone films/tablets on hand in case they are needed.
2. Scheduling regular telephone or in-person follow-up visits to monitor for cravings, withdrawal symptoms, or other potential relapse triggers.
3. Ensuring naloxone is readily available in the home for emergency use.

By proactively addressing risks and supporting ongoing engagement, clinicians can help patients navigate tapering process while ensuring patient safety.

## Case 2

Case vignette: 54 year-old cis-gender male patient with severe OUD in sustained remission presents to your clinic as a regular follow-up visit. He has been on buprenorphine 8 mg films three times a day and has not returned to use for the past 5 years. His urine drug screening is consistently positive for buprenorphine and negative with opioid. Over the treatment course, he became stable with his housing and employment, and started to feel that he is ready to taper off buprenorphine. He is willing to discuss the approach to discontinue his buprenorphine-naloxone treatment.

This patient has been stable in his recovery medically and socially with stable housing and a strong support network. He cannot remember the last time he experienced a craving and is committed to his recovery. Clinicians consider asking the questions listed in Case 1 to help them better understand his circumstances and rationales.

Here are two examples of tapering off buprenorphine-naloxone 8–2 mg film three times a day:

1) Gradually lower the dosage of buprenorphine-naloxone from 8–2 mg film three times a day, 8–2 mg twice a day and then 4–1 mg twice a day. As the dosage becomes lower, patients may find it more difficult to adjust themselves to lower dosage due to craving and withdrawal symptoms. Especially after lowering to buprenorphine 8 mg or lower per day, you can consider a slower tapering plan as below [16, 17].

Step 1. 4 mg/day

Step 2. 2 mg/day

Step 3. 1.5 mg/day

Step 4. 1 mg/day

Step 5. 0.75 mg/day

Step 6. 0.50 mg/day

The tapering interval can vary from a week to several months depending on patient responses to reduced dosage. Throughout the process, carefully individualizing their taper plan is critically important to manage their withdrawal symptoms and cravings. Furthermore, in the tapering process, providing additional doses along with comfort medications is helpful to address cravings and withdrawal symptoms. It is ideal to schedule follow-up visits at intervals that align with the tapering schedule, allowing timely identification of potential issues and prompt adjustment of the treatment plan as needed.

2) Transition to extended-release buprenorphine 300 mg monthly with additional buprenorphine-naloxone as needed, and then 100 mg monthly with additional buprenorphine-naloxone as needed, followed by buprenorphine-naloxone as needed with comfort medications [18, 19]. The dosage of additional buprenorphine-naloxone can range from 2–0.5 mg to 8–2 mg. When clinicians prescribe supplemental doses for a 28-day period (or until their next injection), patients often retain extra doses. This allows them to take an additional dose if needed-particularly after the third week, when the extended-release injection may begin to wear off. The timeline for reducing the extended-release dose can greatly vary among individuals, ranging from one month to several months. It is important to ensure that patients feel comfortable and supported in trying a lower monthly dose. If needed, returning to the 300 mg monthly dose from 100 mg is entirely appropriate and should be framed as a flexible, patient-centered approach.

Both approaches are safe and effective. Clinicians can select either of the two approaches depending on the patient’s preference. If patients have limited or unreliable transportation access to in-person visits, approach (1) with sublingual buprenorphine may be preferable, as it does not require transportation to a clinic.

Continuing to follow up with patients after discontinuing buprenorphine is crucial for detecting any signs of relapse early on and facilitating a seamless transition to opioid antagonist treatment and/or psychosocial interventions. Additionally, it is essential to emphasize that it is completely reasonable to return to the original buprenorphine-naloxone dose if patients encounter challenges with cravings or withdrawal symptoms during their recovery journey.

## Conclusion

The plan of if and how to taper off buprenorphine is most successful when done through shared decision-making. Ideally, clinicians have developed a deep understanding of the patient’s substance use journey and can understand the factors, both internal and external, driving the decision. Through this understanding, a clinician can respect what successful treatment means to each patient. The discussion around tapering allows a unique opportunity to discuss what stability and well-being look like to the patient through the concept of recovery capital [13]. Subsequently, the patient and clinician can develop a plan based on their well-being, safety, and risk factors for return to use. For some patients, this may lead to the decision to continue current treatment rather than taper. Others may integrate information from the clinician with their own treatment history and decide to taper.

In our practice, we have found a few helpful tools that help taper safely and successfully. As discussed previously, we develop a deep understanding of why the patient wants to taper and what their expectations are around the process. Next, we develop a shared language around what stability means in their recovery journey. We utilize an adaptation of the concept of recovery capital to help assess stability and well-being at each visit [13]. This concept reviews areas in a person’s life that are supporting recovery and can be protective as they taper off buprenorphine. Conversely, it allows for the identification of risk factors for return to use, such as life stressors or a new physical health diagnosis.

Finally, the decision to discontinue buprenorphine can be one of the most important decisions that a clinician can share with a patient. It is essential to highlight that tapering is not a one and done choice. For example, if a patient finds that they are struggling with a dose reduction of buprenorphine and at increased risk of returning to use, the best choice may be to resume the previous stable dose and revise the decision to taper. Most importantly, it is vital to reinforce that buprenorphine is a life-saving medication, can be continued life-long, and is a tool for recovery, not a barrier.

## Data Availability

No datasets were generated or analysed during the current study.

## Abbreviations

OUD: Opioid use disorder

## References

[1] Substance Abuse and Mental Health Services Administration. Key substance use and mental health indicators in the United States: Results from the 2024 National Survey on Drug Use and Health. Published July 2025. https://www.samhsa.gov/data/data-we-collect/nsduh-national-survey-drug-use-and-health/national-releases. Accessed July 28, 2025.

[2] Spencer MR, Garnett MF, Miniño AM. Drug overdose deaths in the United States, 2002–2022. NCHS Data Brief, no 491. Hyattsville, MD: National Center for Health Statistics. 2024.

[3] Larochelle MR, Bernson D, Land T, et al. Medication for opioid use disorder after nonfatal opioid overdose and association with mortality: a cohort study. Ann Intern Med. 2018;169(3):137. 10.7326/M17-3107. 29913516 10.7326/M17-3107 PMC6387681

[4] Pearce LA, Min JE, Piske M, et al. Opioid agonist treatment and risk of mortality during opioid overdose public health emergency: population based retrospective cohort study. BMJ. 2020;368:m772. 10.1136/bmj.m772. 32234712 10.1136/bmj.m772 PMC7190018

[5] MacArthur GJ, Minozzi S, Martin N, et al. Opiate substitution treatment and HIV transmission in people who inject drugs: systematic review and meta-analysis. BMJ. 2012;345:e5945. 10.1136/bmj.e5945. 23038795 10.1136/bmj.e5945 PMC3489107

[6] Barocas JA, Gai MJ, Amuchi B, Jawa R, Linas BP. Impact of medications for opioid use disorder among persons hospitalized for drug use-associated skin and soft tissue infections. Drug Alcohol Depend. 2020;215:108207. 10.1016/j.drugalcdep.2020.108207. 32795883 10.1016/j.drugalcdep.2020.108207 PMC7502512

[7] Volkow ND. Stigma and the toll of addiction. N Engl J Med. 2020;382(14):1289–90. 10.1056/NEJMp1917360. 32242351 10.1056/NEJMp1917360

[8] Monico LB, Gryczynski J, Mitchell SG, Schwartz RP, O’Grady KE, Jaffe JH. Buprenorphine treatment and 12-step meeting attendance: conflicts, compatibilities, and patient outcomes. J Subst Abuse Treat. 2015;57:89–95. 10.1016/j.jsat.2015.05.005. 25986647 10.1016/j.jsat.2015.05.005 PMC4560966

[9] Hazle MC, Saxon AJ, Hill KP. Buprenorphine in safety-sensitive positions. Am J Drug Alcohol Abuse. 2022;48(3):255–9. 10.1080/00952990.2021.2003809. 35030309 10.1080/00952990.2021.2003809

[10] US. Department of Justice Civil Rights Division. ADA.gov. Accessed July 28, 2025. https://www.ada.gov

[11] Glanz JM, Binswanger IA, Clarke CL, et al. The association between buprenorphine treatment duration and mortality: a multi-site cohort study of people who discontinued treatment. Addiction. 2023;118(1):97–107. 10.1111/add.15998. 35815386 10.1111/add.15998 PMC9722535

[12] Weimer MB, Herring AA, Kawasaki SS, Meyer M, Kleykamp BA, Ramsey KS. ASAM clinical considerations: buprenorphine treatment of opioid use disorder for individuals using high-potency synthetic opioids. J Addict Med. 2023;17(6):632–9. 10.1097/ADM.0000000000001202. 37934520 10.1097/ADM.0000000000001202

[13] Zweben JE, Sorensen JL, Shingle M, Blazes CK. Discontinuing methadone and buprenorphine: a review and clinical challenges. J Addict Med. 2021;15(6):454–60. 10.1097/ADM.0000000000000789. 33323695 10.1097/ADM.0000000000000789 PMC10082633

[14] Bowen E, Irish A, LaBarre C, Capozziello N, Nochajski T, Granfield R. Qualitative insights in item development for a comprehensive and inclusive measure of recovery capital. Addict Res Theory. 2022;30(6):403–13. 10.1080/16066359.2022.2055002. 36721868 10.1080/16066359.2022.2055002 PMC9886235

[15] Vilsaint CL, Kelly JF, Bergman BG, Groshkova T, Best D, White W. Development and validation of a brief assessment of recovery capital (BARC-10) for alcohol and drug use disorder. Drug Alcohol Depend. 2017;177:71–6. 10.1016/j.drugalcdep.2017.03.022. 28578224 10.1016/j.drugalcdep.2017.03.022

[16] Kumar R, Viswanath O, Saadabadi A. Buprenorphine. In: StatPearls [Internet]. Treasure Island, FL: StatPearls Publishing; 2025 Jan-. Updated June 8, 2024. Available from: https://www.ncbi.nlm.nih.gov/books/NBK459126/29083570

[17] ReVIDA Recovery. How to Taper Off Suboxone. March 7, 2022. https://www.revidarecovery.com/news/how-to-taper-off-suboxone/#:~:text=An%20additional%20rule%20is%20that,of%20buprenorphine%20(Suboxone®)

[18] Ritvo AD, Calcaterra SL, Ritvo JI. Using extended release buprenorphine injection to discontinue sublingual buprenorphine: a case series. J Addict Med. 2021;15(3):252–4. 10.1097/ADM.0000000000000738. 32925232 10.1097/ADM.0000000000000738

[19] Epland C, Pals H, Hayden J. Buprenorphine enhanced taper tolerability evaluation report (BETTER): a case series. Subst Use Addict J. 2024. 10.1177/29767342241242242. 10.1177/29767342241242242 38591225

